# Liu Shen Capsule Alters Airway Microbiota Composition and Metabolite Profiles in Healthy Humans

**DOI:** 10.3389/fphar.2021.824180

**Published:** 2022-01-28

**Authors:** Xuerui Wang, Xiaolong Xu, Yishan Chen, Zhenxuan Li, Mina Zhang, Chunxia Zhao, Bo Lian, Jingxia Zhao, Yuhong Guo, Qingquan Liu

**Affiliations:** ^1^ Beijing Hospital of Traditional Chinese Medicine, Capital Medical University, Beijing, China; ^2^ Beijing Key Laboratory of Basic Research with Traditional Chinese Medicine on Infectious Diseases, Beijing, China; ^3^ Beijing Institute of Chinese Medicine, Beijing, China

**Keywords:** airway microbiota, circulating metabolite, fecal metabolite, Liu Shen capsule, traditional Chinese medicine

## Abstract

Alteration in airway microbiota composition and perturbations in microbe-metabolites interactions have been proposed as markers of many diseases. Liu Shen (LS) capsule, a traditional Chinese medicine, was proved as favorable in treating respiratory diseases. However, the effects of the LS capsule in terms of regulating human microorganisms and metabolite profiles are not well known. This study aimed to define and compare the respiratory microbiota composition and circulating and fecal metabolite profiles before and after LS capsule administration. A total of 30 healthy volunteers were recruited. The pharyngeal swab samples were collected for 16S rRNA gene sequencing. The serum and fecal samples were collected to analyze the non-targeted ultra-performance liquid chromatography–tandem mass spectrometry metabolomics. The airway microbial compositions were profoundly altered after LS capsule administration, as evidenced by increased microbial diversity and altered microbial taxa distribution. The increasing abundance of bacterial *Bifidobacteria*, and *Lactobacillus* characterized the after-administration groups, and the increasing of abundance bacterial Proteobacteria*, Veillonella*, *Prevotella*, *Neisseria*, and *Actinomyces* characterized the before-administration groups. Significant discriminations were observed in both serum and fecal metabolic profiles between the before- and after-administration groups. A total number of 134 and 71 significant HMDB taxonomic metabolites including glycerophospholipids, fatty acyls, and prenol lipids in the serum and fecal samples were identified respectively between the before- and after-administration groups. The integrated analysis showed that some altered airway microbiota phylum, such as Bacteroidetes and Proteobacteria, significantly correlated with metabolites in serum and fecal. Hence, our study reported the alternations in the composition and functions of the airway microbial community and the changes in circulating and fecal metabolite profiles after LS capsule administration in healthy humans, thus providing a novel insight into the mechanisms underlying the role of LS capsule treating and preventing related diseases.

## Introduction

The respiratory microbiota plays an essential role in the development, education of the immune system, and maintenance of the homeostasis. Antimicrobial defense is impaired in germ-free and microbiota-depleted animals, leading to a high possibility of respiratory infections (Maschirow et al., 2019). Previous studies also found shrunk lungs and less mature alveoli development in germ-free rodents (Yun et al., 2014). Microbes and their products tune the immune system toward healthy homeostasis and provide local and systemic signals to the immune system to support the protective responses against diverse pathogens. The pivotal role of airway microbiota has been pointed out by clinical and basic studies in various diseases, for example pulmonary hypertension, influenza, 30445563 and chronic obstructive pulmonary disease (COPD) (Ramos-Sevillano et al., 2019; Zhang et al., 2020a; Wang et al., 2021). Linked by a key mediator, the immune system, the microbiome-host interaction influences the clinical outcomes. The respiratory tract microbiome in asthma patients is associated with T-helper-17 (Th17) cell regulated inflammatory responses and disease severity (Huang et al., 2015). In sepsis and the acute respiratory distress syndrome patients, ltered lung microbiota was significantly correlated with alveolar TNF-α amd systemic inflammatory response (Dickson et al., 2016). The gene expression profile analysis in lung transplant recipients also distinguishes a neutrophilic activation profile pattern (Firmicutes or Proteobacteria colonization dominant) from a macrophage-dominant remodeling profile pattern (Bacteroidetes colonization dominant) (Bernasconi et al., 2016). In mice, lung bacterial composition are correlated with lung concentrations of interleukin (IL)-1α and IL-4, which does not alter after the usage of IL-1 receptor blockade; supporting the concept that microbiota drives the immune phenotype (Dickson et al., 2018).

Over the years, accumulating evidence has identified metabolites produced by respiratory microbiota that can influence host immunity (Man et al., 2017; Budden et al., 2019). The metabolic by-products derived from bacterial fermentation have been reported as the key local and systemic signaling molecules in sustaining immune and tissue homeostasis (Buck et al., 2017). For instance, oral microbiomes produce vasoactive and anti-inflammatory nitrite, nitric oxide, and other bioactive nitrogen oxides (Koch et al., 2017; Pignatelli et al., 2020). Moreover, specific microbiota-associated metabolites, such as short-chain fatty acids (SCFAs), have been shown to affect the progression of varied diseases, including respiratory diseases (Hecker et al., 2021; Zhang et al., 2021). Lung microbiota comprise nitrate reducers and SCFAs producers, such as *Pseudomonas* species and *Staphylococcus* species, which are linked to protection against respiratory diseases by inhibiting histone deacetylases or binding GPR41, GPR43, and GPR109A to alter chemotaxis and phagocytosis, change cell proliferation, and regulate inflammatory responses (McKenzie et al., 2017).

Liu Shen (LS) capsule is a traditional Chinese medicine first prescribed in Qing dynasty. It is widely used in China for treating influenza, tonsillitis, pharyngitis, and mumps (Liu et al., 2018; Wang et al., 2020). The composition of LS capsule includes bezoar (the gallstone of *Bos taurus domesticus* Gmelin), musk (the excretion of *Moschus*), cinobufagin venom toad (the excretion of Venenum Bufonis), pearl (the shell of Pernulo), realgar, and borneol. A number of studies have shown that LS displayed an anti-inflammatory, anti-cancer, anti-viral, analgesic, and anti-bacterial activities in a variety of diseases. In treating influenza, LS capsule inhibited the virus replication and proliferation *in vitro* and ameliorated pneumonia damage *in vivo* via suppressing the TLR4/NF-кB signaling pathway (Ma et al., 2020a; Zhao et al., 2021). LS capsule also inhibited SARS-CoV-2 virus infection via regulating the activity of the NF-κB/MAPK signaling pathway *in vitro* (Ma et al., 2020b). These studies suggested the potential effect of LS capsule on respiratory diseases. However, little is known about the effect of LS capsule on microbiota and metabolite profiles. Therefore, in this study, we used 16S ribosomal RNA (16S rRNA) gene sequencing and metabolomics to systematically characterize altered airway microbial communities and circulating and fecal metabolism, and to further analyze their potential interactions in healthy individuals after LS capsule administration.

## Materials and Methods

### Study Design and Intervention

Thirty healthy volunteers were recruited through advertisements online at Beijing Hospital of Traditional Chinese Medicine, China, between November 2 and 20, 2020. The inclusion criteria were as follows: age 18–45 years; male body weight ≥50 kg, female body weight ≥45 kg, and body mass index (BMI) in the range of 19.0–26.0 kg/m^2^. The participants had no history of chronic or serious diseases in cardiovascular, liver, kidney, respiratory, blood and lymph, endocrine, immune, mental, neurological, gastrointestinal, and other systems. They were able to communicate well with the researchers and agreed to sign the informed consent form. The exclusion criteria were as follows: antibiotics used in the recent 2 months, a history of gastrointestinal diseases within 3 years (including existing ones), diseases with abnormal clinical manifestations excluded; pregnant or lactating women, constitution allergy or Chinese medicine allergy, and participants in other trials in the recent 3 months.

Healthy participants were given one oral LS capsule three times a day for 7 days. LS capsule was provided by Lei Yun Shang Pharmaceutical Group Co., Ltd (Suzhou; batch number ra18029a).

The study was conducted according to the Declaration of Helsinki and approved by the Ethics Committee of Beijing Hospital of Traditional Chinese Medicine Affiliated to Capital Medical University (No. 2019BL02-047-02). The registration number is ChiCTR2000032794 on chictr.org.cn. All participants gave written informed consent.

### Sample Collection

The pharyngeal swab, morning fasting blood, mid-morning urine, and fecal samples were collected on the first and eighth days of the trial (before and after oral LS capsule administration). The pharyngeal swabs were tested for 16S ribosomal RNA Gene (16S rRNA) sequencing. The samples were collected strictly following the process. All participants were forbidden to brush their teeth, gargle, or eat breakfast before collecting morning swabs. The swab should not touch the teeth, oral cavity, and tongue mucosa. Afterward, the swab head was cut off and inserted into a sterilized cryopreservation tube. The samples were stored at −80°C for further processing. The serum and fecal samples were tested for metabolites using a non-targeted metabolomics ultra-performance liquid chromatography–tandem mass spectrometry (UPLC-MS/MS). The blood samples were kept at room temperature for 30 min for clotting. The clotted blood samples were centrifuged at 3000 *g* at 4°C for 20 min to remove the supernatant serum and quickly stored at −80°C for further processing. The fecal samples were collected in a sterile conical tube and immediately frozen at −80°C until further analysis. The morning fasting blood and mid-morning urine samples were tested for blood routine, urine routine, liver function, and renal function to evaluate drug safety.

### DNA Extraction and High-Throughput 16S rRNA Sequencing

DNA from pharyngeal swabs was isolated using an E. Z.N.A. soil DNA Kit (Omega Bio-Tek, GA, United States) following the manufacturer’s protocols. Total DNA quality was measured using a spectrophotometer (NanoDrop 2000 UV; Thermo Fisher Scientific, MA, United States) with 1% agarose gel electrophoresis. 16S sequencing was detected by Biomarker Technologies. The V3–V4 hypervariable regions of the bacterial 16S rRNA gene were amplified with the following primer pairs: 338F (5′-ACT​CCT​ACG​GGA​GGC​AGC​AG-3′) and 806R (5′-GGACTACHVGGGTWTCTAAT-3′) using a thermocycler polymerase chain reaction system (ABI GeneAmp 9700, ABI, United States). The polymerase chain reactions were conducted using the following program: 3 min of denaturation at 95°C, 27 cycles of 30 s at 95°C, 30 s for annealing at 55°C, and 45 s for elongation at 72°C, and a final extension at 72°C for 10 min. Polymerase chain reactions were performed in triplicate using a 20-μl mixture containing 4 μl of 5× FastPfu Buffer, 2 μl of 2.5 mmol/L dNTPs, 0.8 μl of each primer (5 μmol/L), 0.4 μl of FastPfu polymerase, 0.2 μl of bovine serum albumin, and 10 ng of template DNA. The yielding polymerase chain reaction products were extracted from a 2% agarose gel and further purified using an AxyPrep DNA Gel Extraction Kit (Axygen Biosciences, CA, United States) and quantified using QuantiFluor (Promega, United States) following the manufacturer’s protocol.

### Sample Preparation and Ultra-performance Liquid Chromatography–Tandem Mass Spectrometry Analysis for Metabolomics

For the serum samples, 100 μl of the samples were mixed with 400 μl of ice-cold methanol/water solution. For the fecal samples, 50 mg samples were weighed and mixed with 400 μl of ice-cold methanol/water solution. The samples were homogenized at 60 Hz for 6 min using a mechanical disruptor. They were vortexed for 15 min, sonicated for 10 min three times, then placed at −20°C for 30 min to precipitate proteins. After centrifugation at 13,000 *g* at 4°C for 15 min, the supernatant was prepared for ultra-performance liquid chromatography–tandem mass spectrometry (UPLC-MS/MS)/MS analysis. UPLC-MS/MS analyses were performed using a ultra-high performance liquid chromatograph system (1290, Agilent Technologies). The mobile phase consisted of 25 mM NH_4_OAc and 25 mM NH_4_OH in water (pH = 9.75) and acetonitrile. The Triple TOF mass spectrometer was used to acquire MS/MS spectra on an information-dependent acquisition basis during an LC/MS experiment. In this mode, the acquisition software (Analyst TF 1.7, AB Sciex) continuously evaluated the full-scan survey MS data as it collected and triggered the acquisition of MS/MS spectra depending on the preselected criteria. In each cycle, 12 precursor ions with intensity greater than 100 were chosen for fragmentation at collision energy (CE) of 30 V (15 MS/MS events with product ion accumulation time of 50 ms each). Mass data were collected in both positive and negative modes. Quality control samples were injected at regular intervals (every 10 samples). All raw data were imported into the Progenesis QI 2.3 (Nonlinear Dynamics, Waters, United States) and SIMCA-P C 14.0 software package for further data analysis.

### Safety and Adverse Event Monitoring

Adverse events were assessed according to the Common Terminology Criteria for Adverse Events version 4.03 on days 1 and 8 of the trial. The laboratory examinations were standardized in the laboratory department of the hospital. On days 1 and 8 of the trial, routine blood examinations for complete blood cell count and serum biochemical tests for renal and liver functions were conducted for the safety evaluation.

### Statistical Analysis

Ace, Chao, and Shannon indices were calculated to assess *a*-diversity. The *ß*-diversity was estimated by computing the Bray-Curtis dissimilarity and visualized using principal coordinate analysis, and the results were plotted using the R software. The nonparametric Wilcoxon test was used to analyze the different taxonomies at the phylum and genus levels. Statistically significant differences in genera between groups were determined using a linear discriminant analysis (LDA) effect size (LEfSe) algorithm. LDA >3.5 with a *p* < 0.05 was considered significantly enriched. For metabolomics analysis, data obtained in the positive ion mode were used for orthogonal partial least squares discriminant analysis (OPLS-DA) algorithms to visually compare metabolite profiles. Through multivariate and univariate analyses, the significant remaining features were identified by database searches, including the Human Metabolome Database (http://www.hmdb.ca) and Kyoto Encyclopedia of Genes and Genomes (KEGG) (http://www.kegg.com). Spearman correlation analysis was used to evaluate the correlations between metabolites and the microbiota. All the data were presented as mean ± standard error of the mean. The significance of the difference between the two groups was analyzed using the Student unpaired-sample *t* test, and multiple comparisons were analyzed using one-way analysis of variance followed by Dunnett’s post hoc test. *p* values were corrected for multiple comparisons using the Benjamini–Hochberg false discovery rate, and *p* < 0.05 indicated a statistically significant difference.

## Results

### Characteristics and Laboratory Tests of the Healthy Volunteers

According to the inclusive and exclusive criteria, samples of 30 healthy volunteers were collected. The characteristic of sex, gender distribution, BMI and smoking status, as well as the laboratory test results before and after LS capsule administration of the healthy volunteers are shown in Table 1. No significant difference were observed in laboratory tests between the before- and after-administration groups. No adverse events were observed before and after administration.

**TABLE 1:** Characteristics and laboratory tests of the healthy volunteers.

Characteristic	Before	After	*p* Value
Female sex, n (%)	17 (56.6%)	17 (56.6%)	-
Age, y	24.4 ± 0.42	24.4 ± 0.42	-
Body Mass Index, kg/m^2^	20.98 ± 0.82	20.98 ± 0.82	-
Smoking status			
Current	2 (0.06%)	2 (0.06%)	-
Former	2 (0.06%)	2 (0.06%)	-
Never	28 (93.3%)	28 (93.3%)	-
Laboratory tests			
White blood cell count,×10^9^/L	6.57 ± 0.50	7.16 ± 0.86	0.57
Red blood cell count,×10^9^/L	4.62 ± 0.15	4.57 ± 0.16	0.84
Neutrophil count,×10^9^/L	4.18 ± 0.38	4.44 ± 0.59	0.72
Lymphocyte count,×10^9^/L	1.87 ± 0.16	2.08 ± 0.26	0.52
Platelet count,×10^9^/L	252.60 ± 11.57	246.90 ± 11.22	0.78
Hemoglobin, g/L	139.8 ± 4.67	138.3 ± 4.87	0.83
Alanine aminotransferase, U/L	22.33 ± 5.61	20.64 ± 4.37	0.81
Aspartate aminotransferase, U/L	19.55 ± 1.84	20.55 ± 1.81	0.47
Blood urea nitrogen, mmol/L	4.57 ± 0.27	4.34 ± 0.21	0.52
Creatinine, mmol/L	65.34 ± 3.67	64.17 ± 3.44	0.82

### LS Capsule Altered Airway Microbiota Structural Diversity

**FIGURE 1:**
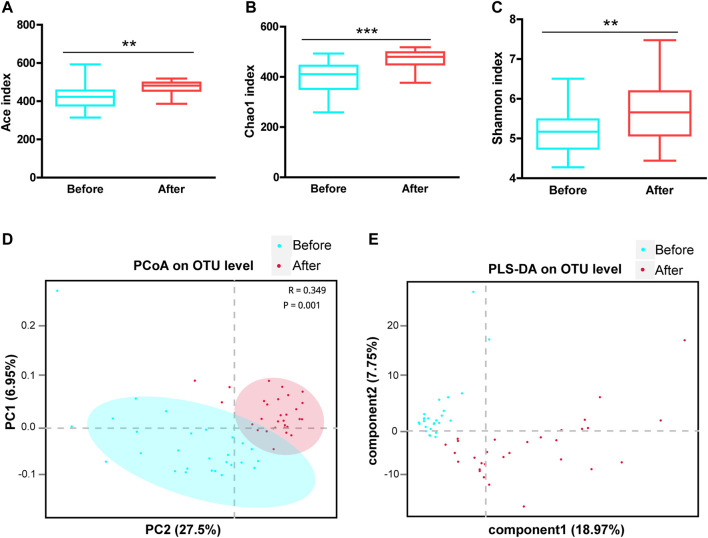
Airway microbial diversity in the before- and after-LS capsule administration groups. *a*-diversity was evaluated based on the Ace **(A)**, Chao1 **(B)**, and Shannon **(C)** indices of the operational taxonomic unit (OTU) levels. ^**^ *p* < 0.01, ^***^ *p* < 0.001. *ß*-diversity was evaluated based on the principal coordinate analysis (PCoA) **(D)** and the partial least squares discriminant analysis (PLS-DA) **(E)** of the OTU levels.

The *a*-diversity indexes, including Ace, Chao1, and Shannon, were used to determine the ecological diversity within a microbial community. These indexes reflected the species richness and evenness, which were significantly higher after LS capsule administration than before administration (Figures 1A–C).

The *ß*-diversity was measured by the principal coordinate analysis (PCoA) (Figure 1D) and the partial least squares discriminant analysis (PLS-DA) (Figure 1E). PCoA based on the Bray-Curtis dissimilarity index showed a significant difference between before- and after-administration groups (Figure 1D, ANOSIM *R* = 0.349, *p* = 0.001). Moreover, PLS-DA represented distinct microbiome profiles before and after LS capsule administration (Figure 1E). Overall, LS capsule administration significantly altered the structural microbial diversity of airways in healthy humans.

### LS Capsule Altered Airway Microbiota Composition

**FIGURE 2:**
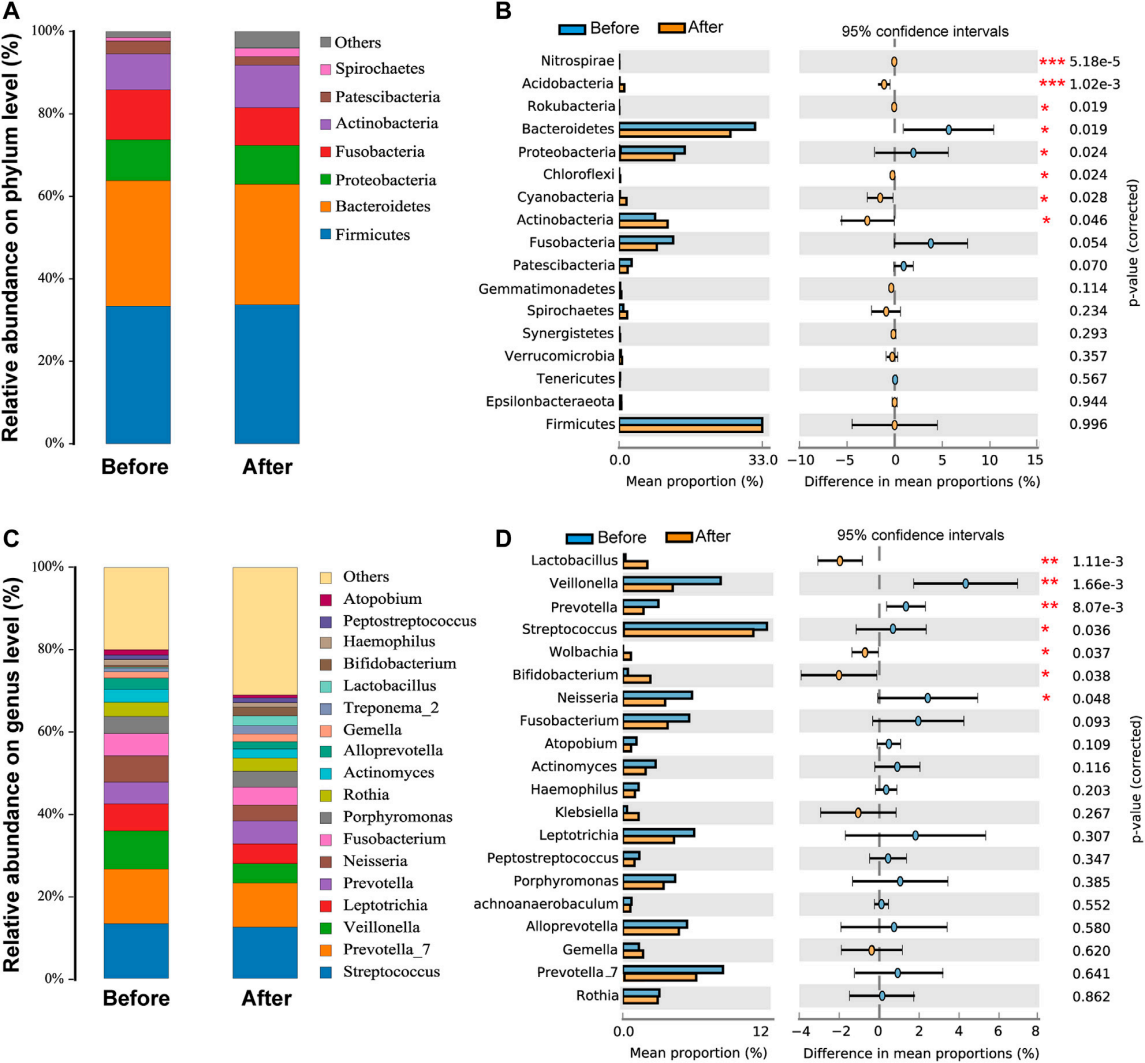
Airway microbiota composition in the before- and after-LS capsule administration groups. The percentage of community abundance at the phylum **(A)** and genus **(C)** levels. Phylum-level **(B)** and genus-level **(D)** bacteria that were significantly different between the before- and after-administration groups. Data were shown as relative abundance (%) of the top 20 most abundant taxa in each group. Statistical analysis was performed using the Wilcoxon rank-sum test. ^*^ *p* < 0.05, ^**^ *p* < 0.01, ^***^ *p* < 0.001.

As shown in Figure 2C, Firmicutes, Bacteroidetes, Proteobacteria, Fusobacteria, Actinobacteria, Patescibacteria, and Spirochaetes were the seven dominant phyla microbiota (relative abundance >1%) in the upper respiratory tract in the before- and after-administration groups (Figure 2A). The proportions of Bacteroidetes, Fusobacteria, and Patescibacteria were smaller after LS capsule administration than those before administration. Mann–Whitney *U* tests were further performed to compare the differences in pharynx bacterial communities between the two groups. At the phylum level, among the top 20 abundant bacterial species, the abundance of Bacteroidetes and Patescibacteria was statistically significantly lower, while the abundance of Nitrospirae, Acidobacteria, Rokubacteria, Chloroflexi, Cyanobacteria, and Actinobacteria was statistically significantly higher, after LS capsule administration than that before administration (Figure 2B).

As shown in Figure 2C, the taxonomic analysis indicated 20 dominant genera (relative abundance >1%) in the before- and after-administration groups. Before LS capsule administration, *Streptococcus* and *Prevotella_7* were the predominant genera (13.3%), followed by *Veillonella* (9.3%). The prevalent genera were *Streptococcus* (12.4%), *Prevotella_7* (10.7%), and *Prevotella* (5.5%) after LS capsule administration. *Prevotella_7*, *Prevotella*, *Leptotrichia*, *Veillonella Fusobacterium*, *Neisseria*, *Actinomyces*, *Alloprevotella*, *Gemella*, *Haemophilus*, *Streptococcus*, and *Atopobium* had a smaller proportion after LS capsule administration than that before administration. At the phylum level, among the top 20 abundant bacterial species, the abundance of *Veillonella*, *Prevotella*, *Streptococcus*, and *Neisseria* was statistically significantly lower after LS capsule administration than that before administration (Figure 2D). In contrast, the abundance of *Lactobacillus*, *Wolbachia*, and *Bifidobacterium* was statistically significantly higher after LS capsule administration than that before administration (Figure 2D).

**FIGURE 3:**
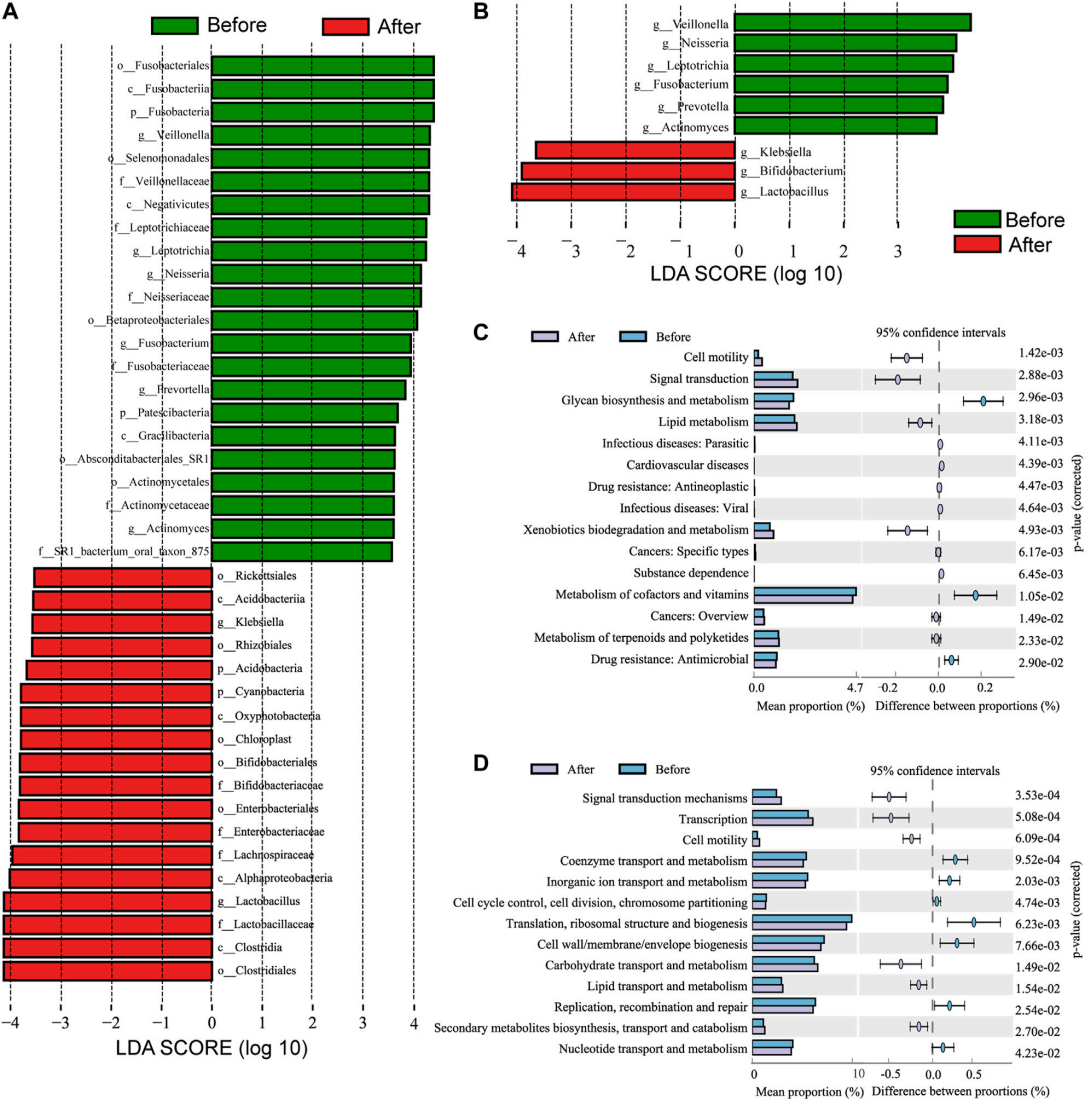
Linear discriminant analysis effect size (LefSe) and linear discriminant analysis (LDA) analysis characterized microbiomes between the before- and after-LS capsule administration groups. **(A)** LDA scores showing the significant bacterial difference between the before- and after-administration groups. Only taxa with LDA scores of >3.5 are presented. Prefix p_ phyla, c_ class, o_ order, f_ family, and g_ genus. **(B)** LDA scores showed a significant bacterial difference between the before- and after-administration groups at the genus level. **(C)** Significant Kyoto Encyclopedia of Genes and Genomes (KEGG) pathway at level 2 and **(D)** Cluster of Orthologous Groups of proteins (COG) at level 2 for the pharynx microbiome in the before- and after-administration groups identified using STAMP software. In STAMP, the differences in abundance between the PH and control groups were analyzed using the White *t* test. Multiple test correction: Benjamini–Hochberg false discovery rate (*p* < 0.05).

Moreover, the differences in microbiota structures from the phylum to the genus level were analyzed. Using a logarithmic LDA score cutoff of 3.5, we identified 40 discriminatory genera as key discriminants (Figure 3A). The abundance of several bacterial including Fusobacteria, *Fusobacteriales*, and *Veillonella* were significantly down-regulated after LS capsule administration, whereas bacterial including Clostridiales*,* Clostridia, and *Lactobacillus* were significantly enriched after LS capsule administration than that before administration. The LDA scoring at the genus level was performed to further dissect the potential roles of the pharynx microbiome in discriminating the before-from after-administration groups (Figure 3B). A higher proportion of *Klebsiella*, *Bifidobacterium*, and *Lactobacillus* was identified in the after-administration group, while a higher proportion of *Veillonella*, *Neisseria*, *Leptotrichia*, *Fusobacterium*, *Prevotella*, and *Actinomyces* was identified in the before-administration group (LDA score [log10] > 3.5).

### LS Capsule Changed the Potential Function of the Airway Microbiome

PICRUSt analysis was carried out to predict the possible impact of the altered airway microbiome by LS capsule administration. The pharynx microbiome with relative abundance greater than 1% was used for analysis. The significantly different abundance compositions of the KEGG pathway (level 2) (Supplementary Table 1) and Cluster of Orthologous Groups of proteins (COG) (level 2) (Supplementary Table 2) between the two groups were listed (*p* < 0.05). As shown in Figure 3C, 12 KEGG pathways, including lipid metabolism and metabolism of terpenoids and polyketides, were significantly enriched after LS capsule administration, while three KEGG pathways, including glycan biosynthesis and metabolism, metabolism of cofactors and vitamins, and drug (antimicrobial) resistance were significantly lower after LS capsule administration. As shown in Figure 3D six functional COG categories were highly enriched after LS capsule administration, including carbohydrate transport and metabolism, lipid transport and metabolism, and secondary metabolite biosynthesis/transport and catabolism. In contrast, seven COG categories, including coenzyme transport and metabolism, inorganic ion transport and metabolism, and nucleotide transport and metabolism, were significantly lower after LS capsule administration. Notably, the dominant COG categories and KEGG pathways associated with LS capsule administration were in the metabolism cluster.

### Alteration of Circulating Metabolome After LS Capsule Administration

**FIGURE 4:**
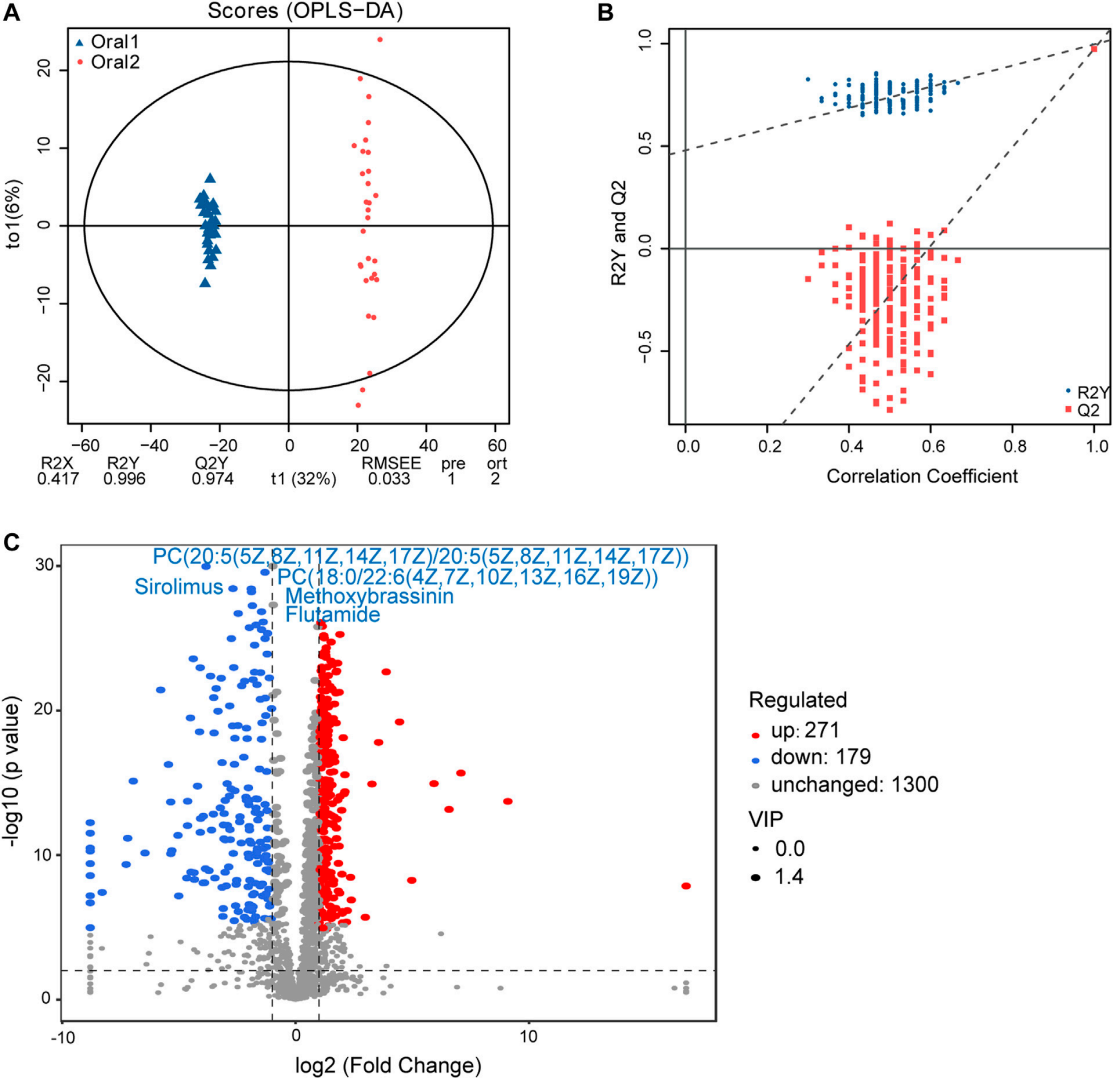
Circulating metabolomics for the quantification of metabolites in the before- and after-administration groups. **(A)** OPLS-DA plots showing the spatial division between the before- and after-LS capsule administration groups. **(B)** Permutation plots of OPLS-DA models correlation coefficient. **(C)** Volcano plot showing the differentially accumulated [log2 (fold-change) on *x*-axis] and significantly changed [–log10 (*p* value) on *y*-axis] metabolites in the before- and after-administration groups.

**FIGURE 5:**
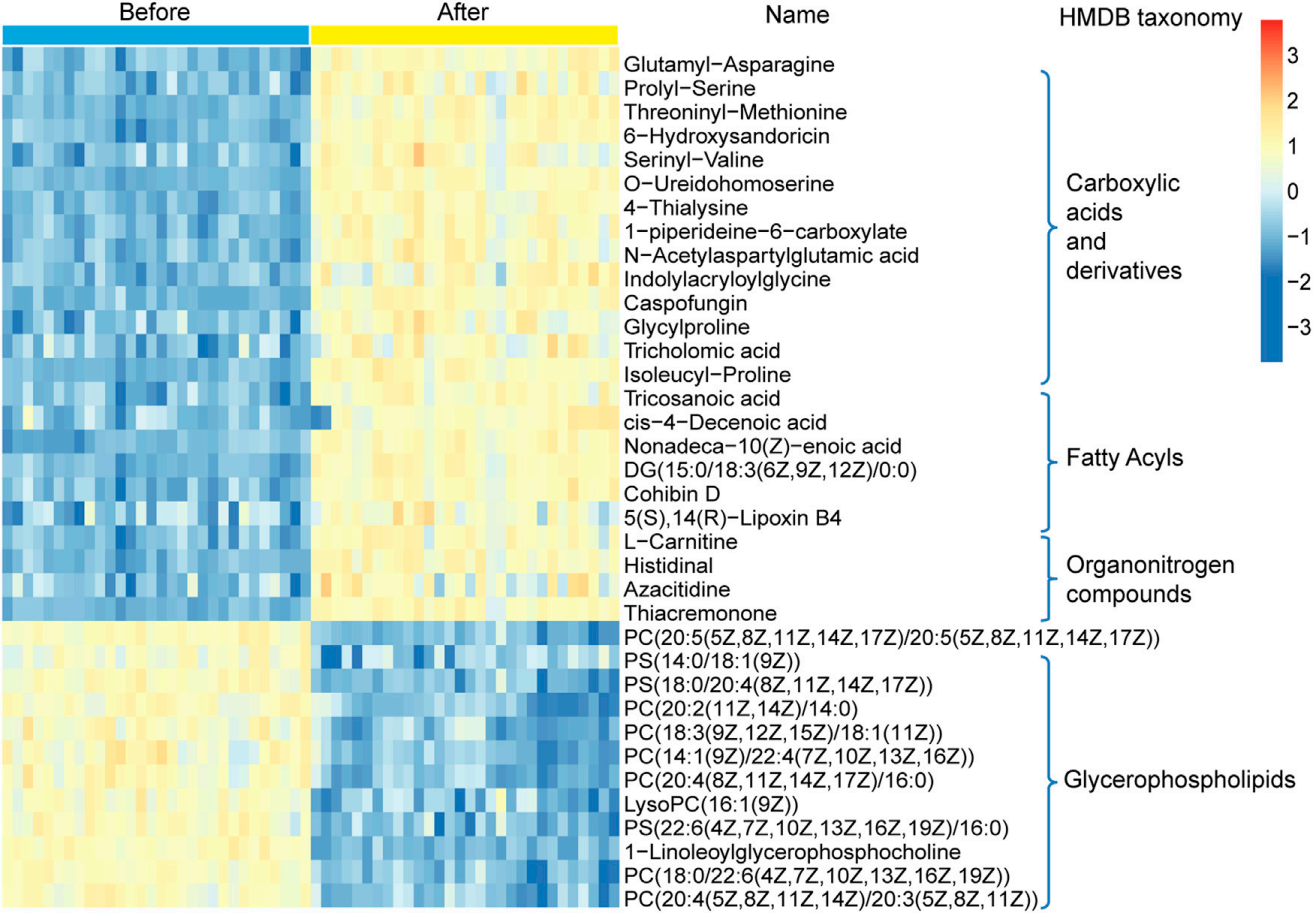
Hierarchical clustering analysis for the circulating metabolites in the before- and after-administration groups based on their z-normalized abundance. The name and HMDB taxonomy clusters of the metabolites were listed.

A non-targeted UPLC-MS/MS metabolomics approach was used to analyze serum samples in the two groups. We successfully quantified 1749 metabolites in the positive ion mode, of which 449 had variable importance in projection (VIP) scores >1 and were significantly different (*p* < 0.05) between the two groups (Supplementary Table 3). The OPLS-DA score plot showed clear discrimination between the two groups with R2X = 0.417, R2Y = 0.996, and Q2Y = 0.974, suggesting that the model was predictive and reliable (Figure 4A). Permutation plots of the correlation coefficients of the two OPLS-DA models verified the validation of the models (Figure 4B). Among the metabolites with significant differences between the two groups, 271 were upregulated and 170 were downregulated. The top five metabolites with the most significant differences included the downregulation of PC(20:5 (5Z,8Z,11Z,14Z,17Z)/20:5 (5Z,8Z,11Z,14Z,17Z)), PC(18:0/22:6 (4Z,7Z,10Z,13Z,16Z,19Z)), sirolimus, methoxybrassinin, and flutamide (Figure 4C). A total of 134 metabolites with HMDB taxonomy are listed in Supplementary Table 4 (Figure 7C). A heat map was constructed to visualize 36 significantly differentially abundant metabolites among them (Figure 5). Carboxylic acids and derivatives, including glutamyl-asparagine, prolyl-serine, threoninyl-methionine, and 6-hydroxysandoricin; fatty acyls, including tricosanoic acid, *cis*-4-decenoic acid, and nonadeca-10(Z)-enoic acid; organonitrogen compounds, including histidinal and azacitidine, showed higher abundance in the after-administration group than in the before-administration group. Glycerophospholipids, including PC(20:5 (5Z,8Z,11Z,14Z,17Z)/20:5 (5Z,8Z,11Z,14Z,17Z)), PS(14:0/18:1 (9Z)), and PS(18:0/20:4 (8Z,11Z,14Z,17Z)), showed lower abundances in the after-administration group than in the before-administration group.

### Alteration of Fecal Metabolome After LS Capsule Administration

**FIGURE 6:**
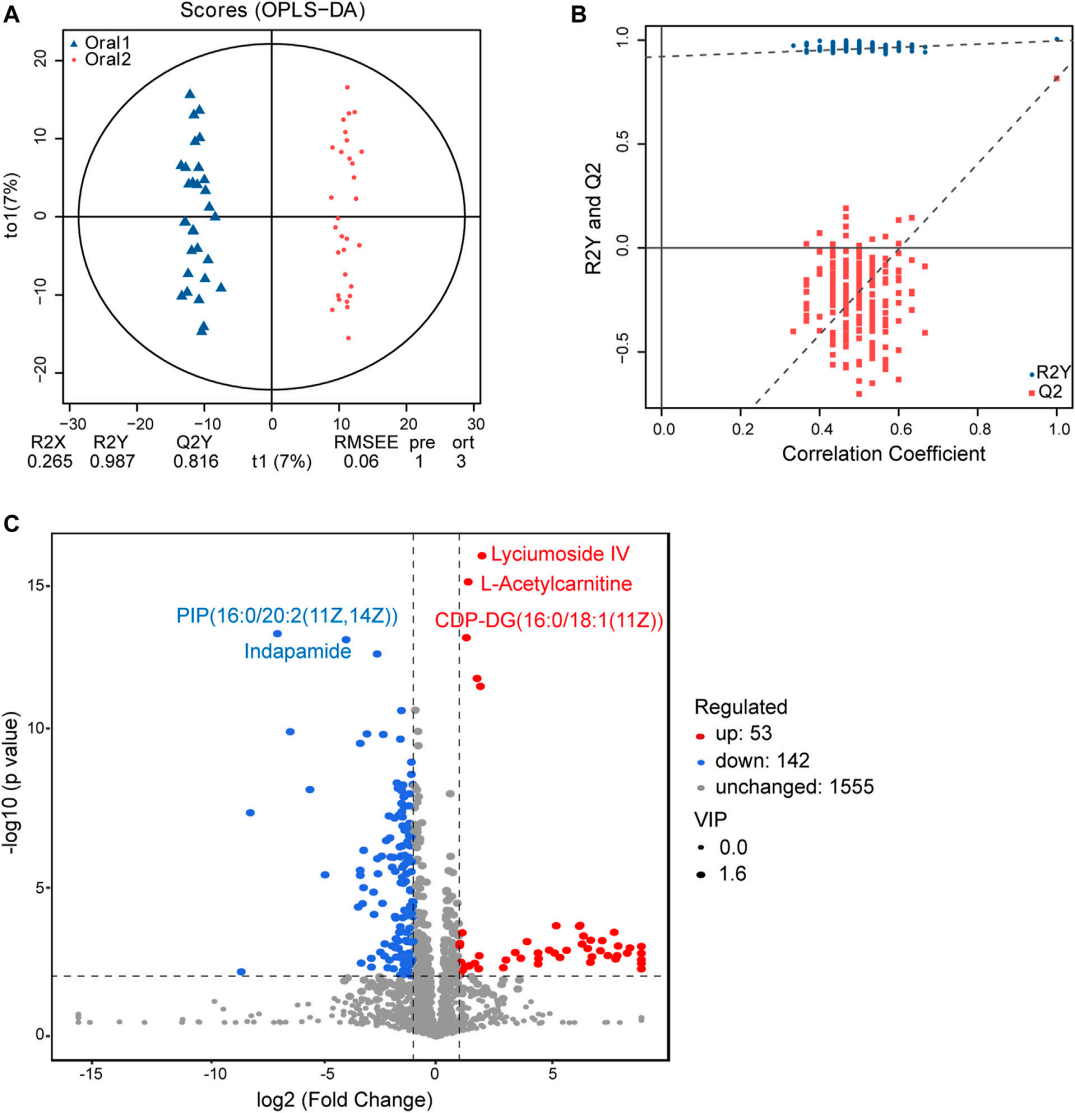
Fecal metabolomics for the quantification of metabolites in the before- and after-administration groups. **(A)** OPLS-DA plots showing the spatial division between the before- and after-LS capsule administration groups. **(B)** Permutation plots of the correlation coefficient of OPLS-DA models. **(C)** Volcano plot showing the differentially accumulated [log2 (fold-change) on *x*-axis] and significantly changed [–log10 (*p* value) on *y*-axis] metabolites in the before- and after-administration groups.

**FIGURE 7:**
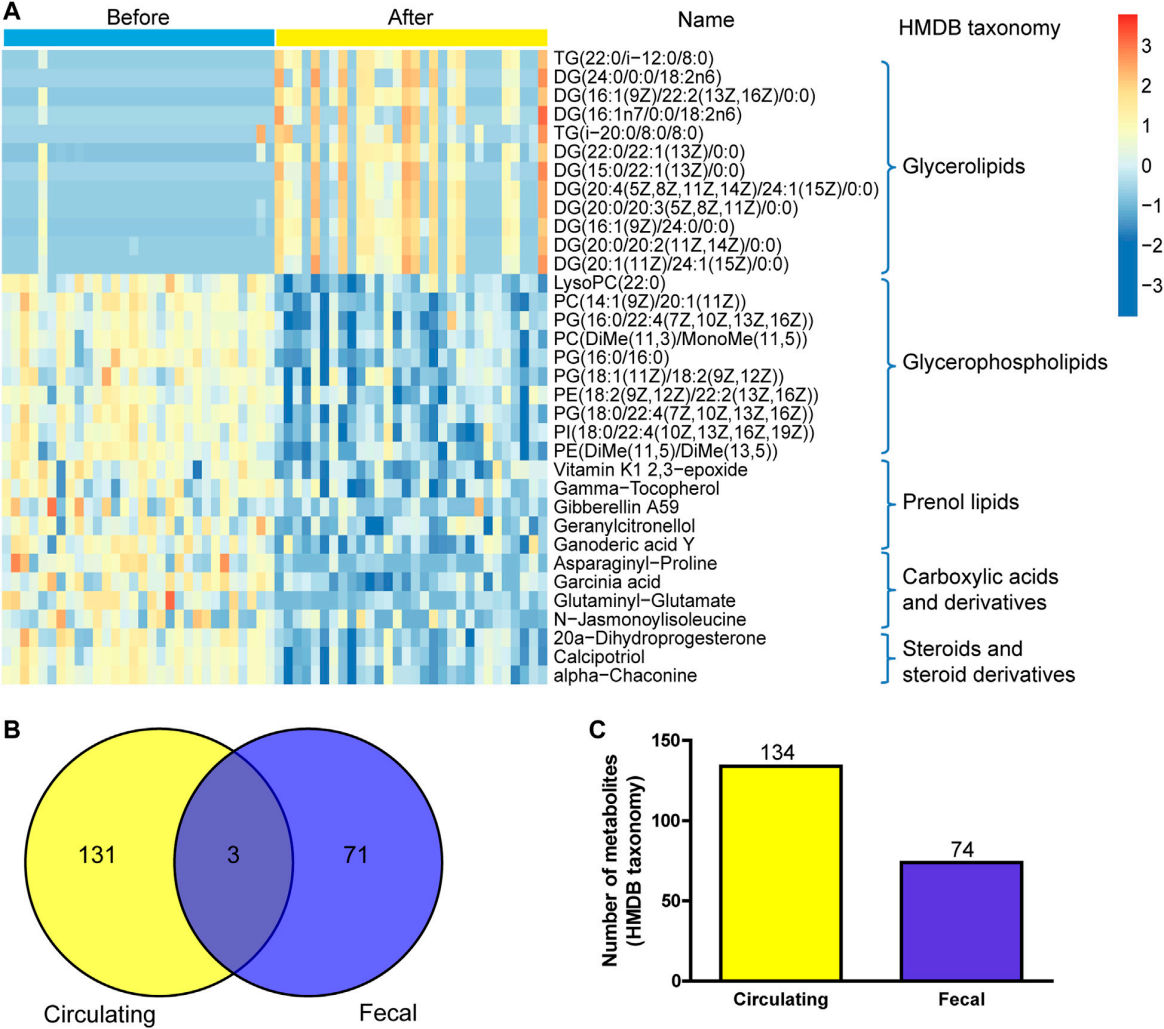
Correlation analysis of fecal metabolites and circulating metabolites. **(A)** Hierarchical clustering analysis for the fecal metabolites in the before- and after-administration groups based on their z-normalized abundances. The name and HMDB taxonomy clusters of the metabolites were listed. **(B)** Venn and number **(C)** of HMDB taxonomy metabolite with differences in serum and fecal samples before and after administration.

Moreover, UPLC-MS/MS metabolomics analysis was used on the fecal samples in the two groups. A total of 1749 metabolites in the positive ion mode were acquired, of which 195 were with VIP scores>1 and were significantly different (*p* < 0.05) between the two groups (Supplementary Table 5). The OPLS-DA score plot showed clear discrimination between two groups with R2X = 0.265, R2Y = 0.987, and Q2Y = 0.816, and the correlation coefficient verified the validation of the models (Figures 6A,B). Among the metabolites with significant differences between the before- and after-administration groups, 53 were upregulated and 143 were downregulated. The top five metabolites with the most significant differences included the downregulation of PIP(16:0/20:2 (11Z,14Z)) and indapamide and the upregulation of lyciumoside IV, L-acetylcarnitine, and CDP-DG (16:0/18:1 (11Z)). A total of 74 metabolites with HMDB taxonomy are listed in Supplementary Table 6 (Figure 7C). A heat map was constructed to visualize 34 significantly differentially abundant metabolites among them (Figure 7). Glycerolipids, including TG (22:0/i-12:0/8:0), DG (24:0/0:0/18:2n6), and DG (16:1 (9Z)/22:2 (13Z,16Z)/0:0), showed higher abundance in the after-administration group than in the before-administration group. Glycerophospholipids, prenol lipids, carboxylic acids and derivatives, and steroids and steroid derivatives showed lower abundance in the after-administration group than in the before-administration group.

134 differential metabolites with HMDB taxonomy in serum samples before and after treatment were compared with 74 differential metabolites with HMDB taxonomy in fecal samples, and three metabolites were overlapped including colistin, gamma-tocopherol, and tetradecanoylcarnitine (Figure 7C). Compared with before administration, the contents of these three metabolites in serum and fecal samples decreased after administration.

### Correlation Analysis of Airway Microbiota and Circulating Metabolic Phenotype

**FIGURE 8:**
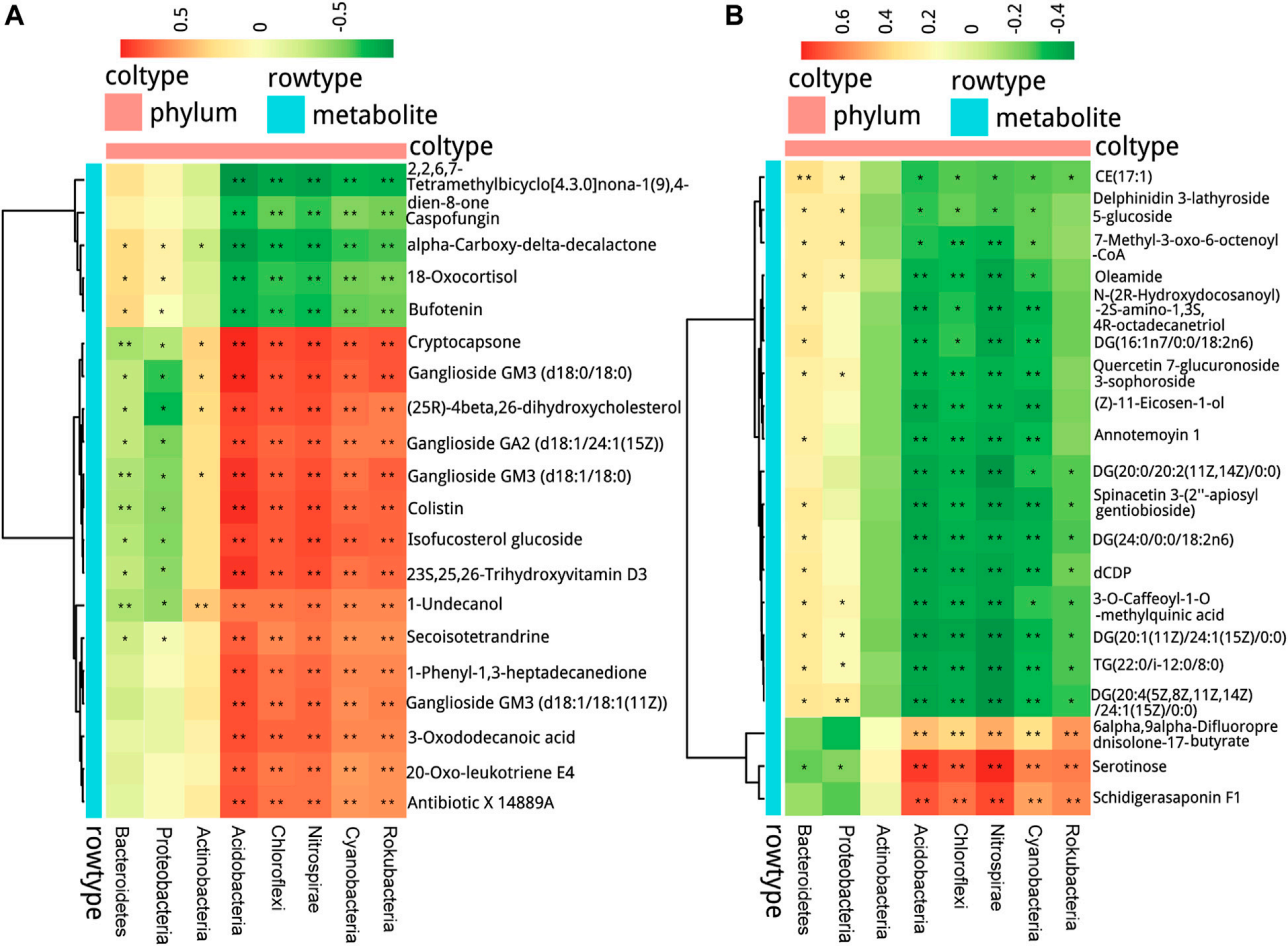
Interrelationship between airway microbiota composition and host metabolic profile. The positive and negative correlations are shown as red and green, respectively, in the heat map according to Spearman correlation analysis between the airway significant microbiota at the phylum level with **(A)** the serum metabolites and **(B)** the fecal metabolites. Significant microbiota–metabolite correlations were presented with ^*^ *p* < 0.05, ^**^ *p* < 0.01, and ^***^ *p* < 0.001.

Spearman correlation analysis was performed between microbial communities at the phylum level and the 20 top fold-change metabolites in serum and fecal samples to explore the functional correlation between pharyngeal microbiota dysbiosis and altered circulating metabolites. In serum samples, as shown in Figure 8A, the abundance of Bacteroidetes and Proteobacteria was positively correlated with the contents of alpha-carboxy-delta-decalactone, 18-oxocortisol, and bufotenin, and negatively correlated with the contents of metabolites including cryptocapsone, ganglioside GM3 (d18:0/18:0), and (25 R)-4beta,26-dihydroxycholesterol in the serum samples. The abundance of Actinobacteria was positively correlated with the contents of metabolites including cryptocapsone, ganglioside GM3 (d18:0/18:0) (25 R)-4beta,26-dihydroxycholesterol, ganglioside GM3 (d18:1/18:1 (11Z)), and 1-undecanol, but negatively correlated with the content of alpha-carboxy-delta-decalactone. The abundance of microbiota Acidobacteria, Chloroflexi, Nitrospirae, Cyanobacteria, and Rokubacteria showed a strong positive correlation with the contents of most of the metabolites including cryptocapsone, ganglioside GM3 (d18:0/18:0), and (25R)-4beta,26-dihydroxycholesterol, and a negative correlation with the contents including 18-oxocortisol and bufotenin.

In fecal samples, as shown in Figure 8B, the abundance of Bacteroidetes and Proteobacteria was positively correlated with the contents of most metabolites including CE (17:1), oleamide, and DG (16:1n7/0:0/18:2n6), and negatively correlated with the content of serotinose. The abundance of Acidobacteria, Chloroflexi, Nitrospirae, Cyanobacteria, and Rokubacteria was positively correlated with the contents of 6alpha,9alpha-difluoroprednisolone-17-butyrate, serotinose, and schidigerasaponin F1, but negatively correlated with the contents of most metabolites including DG (20:1 (11Z)/24:1 (15Z)/0:0) and CE (17:1).

## Discussion

In this study, 16S rRNA sequencing was performed in pharyngeal swab samples, and UPLC-MS/MS-based metabolomics was performed in serum and fecal samples collected from 30 healthy participants before and after LS capsule administration. Our results showed that the respiratory microbiota composition and function and serum/fecal metabolic phenotype were significantly different after LS capsule administration. The potential regulatory mechanism of LS capsule on bacterial microbiota and metabolites might be involved in its prevention or treatment effects on diseases.

The surface area of the human respiratory tract is approximately 40 times larger than that of the skin inhabited by microbiota. For most respiratory pathogens, the colonization of the upper respiratory tract is the first step before causing the infection on the mucosal surface and spreading to the lower respiratory tract (Bogaert et al., 2004). Hence, the microbiota in the upper respiratory tract acts as a “gatekeeper” that provides resistance to pathogen colonization. Clinical studies suggested that the lower respiratory tract microbiota shared considerable similarity to that of the oropharynx in both healthy individuals and patients with chronic lung diseases (Botero et al., 2014). The predominant phyla of healthy individuals are Bacteroidetes and Firmicutes in the lungs and Firmicutes, Proteobacteria, and Actinobacteria in the oropharynx (Pulvirenti et al., 2019; Maleki et al., 2020). The most common genera in the lung and oral cavity are similar, including *Streptococcus*, *Prevotella*, and *Veillonella*, which is consistent with our results. Since the isolation of lower respiratory tract microbiota samples is usually completed by fiberoptic bronchoscopy, which is a risky invasive operation. We selected pharyngeal swab samples and analyzed the effect of LS capsule on the upper respiratory tract microbiota.

The decreased diversity of local bacterial communities is associated with many lung diseases. For example, in patients with cystic fibrosis, a significant ecological pattern with decreasing airway microbiota diversity and a reducing lung function was found (Cuthbertson et al., 2020). In patients and an animal model of pulmonary fibrosis, decreased lung bacterial diversity was significantly associated with increased alveolar concentrations of pro-inflammatory cytokines and growth factors (O’Dwyer et al., 2019). Compared with reference subjects, Zhang et al. also reported decreased community diversity in patients with pulmonary hypertension (Zhang et al., 2020a). Although the causal relationship between microbial diversity and disease occurrence is not clear, these studies demonstrated a certain link between decreased diversity of specific microecological environment and unhealthy outcomes. The traditional Chinese medicine prescription of LS capsule has a history of thousands of years. It is widely used in clinic in China and is effective in treating influenza, acute bronchitis, and other respiratory diseases. The present study found that the diversity of the airway microbiota was significantly increased after LS capsule administration compared with that of before administration. The regulatory effect on microbiota diversity might be one of the potential mechanisms of LS capsule in treating diseases.

LS capsule administration significantly decreased the abundance of several airway bacterial. Among them, Proteobacteria*, Veillonella*, *Streptococcus*, *Prevotella*, *Neisseria* and *Actinomyces* were reported to be highly associated with the development of respiratory diseases. In both upper airway and acellular bronchoalveolar lavage samples from participants without known pulmonary diseases, Segal et al. observed that enriched *Veillonella* and *Prevotella* were associated with the enhanced expression of Th-17 lymphocyte–derived inflammatory cytokines, and conversely, a blunted alveolar macrophage TLR4 response (Segal et al., 2016). Tsay et al. reported enriched airway *Veillonella*, *Prevotella*, and *Streptococcus* in patients with lung cancer, which were associated with the upregulation of the ERK and PI3K signaling pathways and tested in both patients and *in vitro* epithelial cell experiments (Tsay et al., 2018). In patients with asthma, Proteobacteria dominance is associated with hyper-responsiveness and neutrophil-derived excessive airway inflammation (Huang et al., 2011; Yang et al., 2018). In patients with COPD, Proteobacteria dominance was also reported to be associated with increased mortality and neutrophil activation (Dicker et al., 2021). Proteobacteria and Actinobacteria are associated with infiltrating immune cells in lung tissue, including neutrophils, eosinophils, and B cells (Sze et al., 2015). Moreover, increased *Prevotella* abundance was associated with augmented T-helper type 17 (Th17)-mediated mucosal inflammation, which was in line with the marked capacity of *Prevotella* in driving Th17 immune responses *in vitro* (Huang et al., 2020). *Neisseria*, such as *Neisseria meningitidis*, is a colonizing bacterium in the respiratory tract, which can cause diseases under certain conditions. The respiratory mucosa is the site of *Neisseria* colonization and the barrier to protect its invasion (Audry et al., 2019). In our study, we also observed higher taxa of *Bifidobacteria* and *Lactobacillus* after LC capsule administration than before*. Bifidobacteria* and *Lactobacillus* are common probiotics. *Bifidobacterium* was reported to play a protective role on the respiratory mucosa (Flemer et al., 2018). In our study, no pathogenic bacteria (e.g., *S pneumonia*, *H influenzae*) were detected since the participants in both groups were healthy people. Unlike the above bacteria, some altered bacteria, such as *wolbachia*, were barely report related to diseases. Based on the aforementioned findings, we speculated that the mechanism of LS capsule is related to the alternation of the microbiota structure in the upper respiratory tract and the downregulation of certain microbiota closely related to the development of respiratory diseases.

The alternation of respiratory microbiota after LS capsule administration might impact the metabolites, since COG and KEGG pathway analysis of airway microbiota showed several metabolic pathways with signature alternation. Indeed, we confirmed that the serum and fecal samples had completely different metabolomics before and after LS capsule administration. In the serum and the fecal samples of the two groups, the numbers of lipid metabolites were changed the most (50% in the serum and 77% in feces). Lipid is the basic component of the cell membrane and plays an important role in energy storage, signal transduction, and formation of membrane bilayer and cellular barriers. Lipid metabolisms are indicated in numerous human diseases, such as Alzheimer’s disease, respiratory diseases, obesity, and atherosclerosis (Hannun and Obeid, 2018; Zhang et al., 2020b). In our study, we identified several categories of lipid types, including fatty acids, sphingolipids, prenol lipids, and glycerophospholipids. Fatty acyls are a source of energy in cells to produce ATP. We identified the alternation of 16 fatty acyl metabolites between the before- and after-administration groups, of which 5 were downregulated and 11 were upregulated. A growing number of studies proposed fatty acyls as the biomarkers of some diseases. For example, in patients with idiopathic pulmonary fibrosis, 20 fatty acyls have been listed with significant compared with the controls (Yan et al., 2017). A previous study also found that chiral ibuprofen treatment caused disorders in the metabolism of the brain lipids, including glycerophospholipid and fatty acid metabolism, which affected the composition of biological membranes, inflammatory responses, and cardiovascular and cerebrovascular disease development in zebrafish (Zhang et al., 2020c). Sphingolipids are ubiquitous cellular membrane components implicated in multiple cellular processes including autophagy, apoptosis, differentiation, and cell division. We found that two sphingolipids were downregulated after LS capsule administration in serum samples. Previous studies found that four out of 46 sphingolipids could distinguish the patients with idiopathic pulmonary fibrosis from control subjects (Yan et al., 2017). Glycerophospholipids are important biomolecules that constitute the cytoskeleton of the cell membrane, and also a repository of a large number of bioactive media produced by the reaction of phospholipase (Frisardi et al., 2011). We identified the alternation of 26 glycerophospholipid metabolites between the before- and after-administration groups, of which 18 were downregulated and eight were upregulated. Previous studies demonstrated decreased plasma glycerophospholipid levels in patients with cystic fibrosis (Grothe et al., 2015) and those with pulmonary fibrosis (Yan et al., 2017), implying that glycerophospholipid metabolites could be used as potential biomarkers for the aforementioned lung diseases.

The pharmacodynamic components of LS capsule include bufonidine lactones, cholic acid compounds of bezoar, androgen of musk, volatile components in borneol, and minerals in realgar and pearl, among which the former three are the major ones. We detected that the metabolites including bufotenin and shoyuflavone b were upregulated in serum samples after administration, which was due to the metabolism of LS capsule in the body. The levels of benzene compounds, which are usually produced and degraded by bacterial species, were significantly different before and after administration, suggesting that the bacterial–metabolite interaction was involved in the mechanism of LS capsule (Nguyen and Kim, 2019). After oral administration of LS capsule, some drug active components could be directly absorb, while some other components were with low bioavailability or cannot be absorbed, which need to be reused through the transformation of intestinal flora. Blood metabolites (serum metabolomics) and metabolites transformed via intestinal flora (fecal metabolomics) reach the respiratory tract with circulation and regulate the local microbiota. As supported by our results, we observed a significant correlation between airway microbiota with serum and fecal metabolites, indicating that the perturbations of microbiota were associated with metabolic phenotype alterations. We found three metabolites that were significant altered in both feces and serum samples before and after administration. These metabolites could be used as a marker for taking LS capsule. However, since both groups of metabolites were from healthy participants, the abnormalities in main synthetic or catabolic pathways were not detected, nor were the main classes of microorganism-dependent metabolites, such as SCFAs.

In conclusion, our study demonstrated that *Bifidobacteria* and *Lactobacillus* were significantly enriched in the oropharynx respiratory tract samples after LS capsule administration; on the contrary, the bacterial Proteobacteria*, Veillonella*, *Prevotella*, *Neisseria*, and *Actinomyces* were relatively more abundant before administration. These divergent patterns of microbiota composition before and after administration might provide potential novel insights into the mechanisms of LS capsule. Furthermore, we found significant differences in circulating and fecal metabolic profiles before and after administration, especially the changes in lipid-type categories. Our results hinted that LS capsule might play a preventive role in respiratory diseases by regulating microbiota and metabolites. Although the basic characteristics and laboratory tests between the two groups showed no difference, a few samples in PCoA analysis were still discreted. These individual differences are inevitable, since many factors including diet habits, environmental influence, and genetic backgrounds could affect the microbiome composition. The sensitivity and accuracy of non-targeted metabonomics in detecting lipid contents are limited compared to lipid metabolomics. Most importantly, since the participants in both groups were healthy volunteers, whether LS capsule could play similar regulatory effect on respiratory microbiota under respiratory diseases conditions needs to be further investigated in the future.

## Data Availability

The original contributions presented in the study are included in the article/Supplementary Material, further inquiries can be directed to the corresponding author.

## References

[B1] Audry M. Robbe-Masselot C. Barnier J. P. Gachet B. Saubaméa B. Schmitt A. (2019). Airway Mucus Restricts neisseria Meningitidis Away from Nasopharyngeal Epithelial Cells and Protects the Mucosa from Inflammation. mSphere 4, e00494–19. 10.1128/mSphere.00494-19 31801841 PMC6893211

[B2] Bernasconi E. Pattaroni C. Koutsokera A. Pison C. Kessler R. Benden C. (2016). Airway Microbiota Determines Innate Cell Inflammatory or Tissue Remodeling Profiles in Lung Transplantation. Am. J. Respir. Crit. Care Med. 194, 1252–1263. 10.1164/rccm.201512-2424OC 27248293

[B3] Bogaert D. De Groot R. Hermans P. W. (2004). Streptococcus Pneumoniae Colonisation: The Key to Pneumococcal Disease. Lancet Infect. Dis. 4, 144–154. 10.1016/S1473-3099(04)00938-7 14998500

[B4] Botero L. E. Delgado-Serrano L. Cepeda M. L. Bustos J. R. Anzola J. M. Del Portillo P. (2014). Respiratory Tract Clinical Sample Selection for Microbiota Analysis in Patients with Pulmonary Tuberculosis. Microbiome 2, 29. 10.1186/2049-2618-2-29 25225609 PMC4164332

[B5] Buck M. D. Sowell R. T. Kaech S. M. Pearce E. L. (2017). Metabolic Instruction of Immunity. Cell 169, 570–586. 10.1016/j.cell.2017.04.004 28475890 PMC5648021

[B6] Budden K. F. Shukla S. D. Rehman S. F. Bowerman K. L. Keely S. Hugenholtz P. (2019). Functional Effects of the Microbiota in Chronic Respiratory Disease. Lancet Respir. Med. 7, 907–920. 10.1016/S2213-2600(18)30510-1 30975495

[B7] Cuthbertson L. Walker A. W. Oliver A. E. Rogers G. B. Rivett D. W. Hampton T. H. (2020). Lung Function and Microbiota Diversity in Cystic Fibrosis. Microbiome 8, 45. 10.1186/s40168-020-00810-3 32238195 PMC7114784

[B8] Dicker A. J. Huang J. T. J. Lonergan M. Keir H. R. Fong C. J. Tan B. (2021). The Sputum Microbiome, Airway Inflammation, and Mortality in Chronic Obstructive Pulmonary Disease. J. Allergy Clin. Immunol. 147, 158–167. 10.1016/j.jaci.2020.02.040 32353489

[B9] Dickson R. P. Erb-Downward J. R. Falkowski N. R. Hunter E. M. Ashley S. L. Huffnagle G. B. (2018). The Lung Microbiota of Healthy Mice Are Highly Variable, Cluster by Environment, and Reflect Variation in Baseline Lung Innate Immunity. Am. J. Respir. Crit. Care Med. 198, 497–508. 10.1164/rccm.201711-2180OC 29533677 PMC6118022

[B10] Dickson R. P. Singer B. H. Newstead M. W. Falkowski N. R. Erb-Downward J. R. Standiford T. J. (2016). Enrichment of the Lung Microbiome with Gut Bacteria in Sepsis and the Acute Respiratory Distress Syndrome. Nat. Microbiol. 1, 16113. 10.1038/nmicrobiol.2016.113 27670109 PMC5076472

[B11] Flemer B. Warren R. D. Barrett M. P. Cisek K. Das A. Jeffery I. B. (2018). The Oral Microbiota in Colorectal Cancer Is Distinctive and Predictive. Gut 67, 1454–1463. 10.1136/gutjnl-2017-314814 28988196 PMC6204958

[B12] Frisardi V. Panza F. Seripa D. Farooqui T. Farooqui A. A. (2011). Glycerophospholipids and Glycerophospholipid-Derived Lipid Mediators: A Complex Meshwork in Alzheimer's Disease Pathology. Prog. Lipid Res. 50, 313–330. 10.1016/j.plipres.2011.06.001 21703303

[B13] Grothe J. Riethmüller J. Tschürtz S. M. Raith M. Pynn C. J. Stoll D. (2015). Plasma Phosphatidylcholine Alterations in Cystic Fibrosis Patients: Impaired Metabolism and Correlation with Lung Function and Inflammation. Cell Physiol Biochem 35, 1437–1453. 10.1159/000373964 25791258

[B14] Hannun Y. A. Obeid L. M. (2018). Sphingolipids and Their Metabolism in Physiology and Disease. Nat. Rev. Mol. Cel Biol 19, 175–191. 10.1038/nrm.2017.107 PMC5902181 29165427

[B15] Hecker M. Sommer N. Mayer K. (2021). Assessment of Short- and Medium-Chain Fatty Acids on Mitochondrial Function in Severe Inflammation. Methods Mol. Biol. 2277, 125–132. 10.1007/978-1-0716-1270-5_8 34080148

[B16] Huang Y. J. Nariya S. Harris J. M. Lynch S. V. Choy D. F. Arron J. R. (2015). The Airway Microbiome in Patients with Severe Asthma: Associations with Disease Features and Severity. J. Allergy Clin. Immunol. 136, 874–884. 10.1016/j.jaci.2015.05.044 26220531 PMC4600429

[B17] Huang Y. J. Nelson C. E. Brodie E. L. Desantis T. Z. Baek M. S. Liu J. (2011). Airway Microbiota and Bronchial Hyperresponsiveness in Patients with Suboptimally Controlled Asthma. J. Allergy Clin. Immunol. 127, 372–373. 10.1016/j.jaci.2010.10.048 21194740 PMC3037020

[B18] Huang Y. Tang J. Cai Z. Zhou K. Chang L. Bai Y. (2020). Prevotella Induces the Production of Th17 Cells in the colon of Mice. J. Immunol. Res. 2020, 9607328. 10.1155/2020/9607328 33204736 PMC7657696

[B19] Koch C. D. Gladwin M. T. Freeman B. A. Lundberg J. O. Weitzberg E. Morris A. (2017). Enterosalivary Nitrate Metabolism and the Microbiome: Intersection of Microbial Metabolism, Nitric Oxide and Diet in Cardiac and Pulmonary Vascular Health. Free Radic. Biol. Med. 105, 48–67. 10.1016/j.freeradbiomed.2016.12.015 27989792 PMC5401802

[B20] Liu J. Wei L. X. Wang Q. Lu Y. F. Zhang F. Shi J. Z. (2018). A Review of Cinnabar (Hgs) And/or Realgar (As4s4)-containing Traditional Medicines. J. Ethnopharmacol 210, 340–350. 10.1016/j.jep.2017.08.037 28864167

[B21] Ma Q. Huang W. Zhao J. Yang Z. (2020). Liu Shen Wan Inhibits Influenza a Virus and Excessive Virus-Induced Inflammatory Response via Suppression of TLR4/NF-Κb Signaling Pathway *In Vitro* and *In Vivo* . J. Ethnopharmacol 252, 112584. 10.1016/j.jep.2020.112584 31972325

[B22] Ma Q. Pan W. Li R. Liu B. Li C. Xie Y. (2020). Liu Shen Capsule Shows Antiviral and Anti-inflammatory Abilities against Novel Coronavirus SARS-CoV-2 via Suppression of NF-Κb Signaling Pathway. Pharmacol. Res. 158, 104850. 10.1016/j.phrs.2020.104850 32360580 PMC7192119

[B23] Maleki A. Zamirnasta M. Taherikalani M. Pakzad I. Mohammadi J. Krutova M. (2020). The Characterization of Bacterial Communities of Oropharynx Microbiota in Healthy Children by Combining Culture Techniques and Sequencing of the 16s Rrna Gene. Microb. Pathog. 143, 104115. 10.1016/j.micpath.2020.104115 32135220

[B24] Man W. H. de Steenhuijsen Piters W. A. Bogaert D. (2017). The Microbiota of the Respiratory Tract: Gatekeeper to Respiratory Health. Nat. Rev. Microbiol. 15, 259–270. 10.1038/nrmicro.2017.14 28316330 PMC7097736

[B25] Maschirow L. Suttorp N. Opitz B. (2019). Microbiota-dependent Regulation of Antimicrobial Immunity in the Lung. Am. J. Respir. Cel Mol Biol 61, 284–289. 10.1165/rcmb.2019-0101TR 31059654

[B26] McKenzie C. Tan J. Macia L. Mackay C. R. (2017). The Nutrition-Gut Microbiome-Physiology axis and Allergic Diseases. Immunol. Rev. 278, 277–295. 10.1111/imr.12556 28658542

[B27] Nguyen T. M. Kim J. (2019). Sphingobium Aromaticivastans Sp. Nov., a Novel Aniline- and Benzene-Degrading, and Antimicrobial Compound Producing Bacterium. Arch. Microbiol. 201, 155–161. 10.1007/s00203-018-1611-2 30560286

[B28] O'Dwyer D. N. Ashley S. L. Gurczynski S. J. Xia M. Wilke C. Falkowski N. R. (2019). Lung Microbiota Contribute to Pulmonary Inflammation and Disease Progression in Pulmonary Fibrosis. Am. J. Respir. Crit. Care Med. 199, 1127–1138. 10.1164/rccm.201809-1650OC 30789747 PMC6515865

[B29] Pignatelli P. Fabietti G. Ricci A. Piattelli A. Curia M. C. (2020). How Periodontal Disease and Presence of Nitric Oxide Reducing Oral Bacteria Can Affect Blood Pressure. Int. J. Mol. Sci. 21, 21. 10.3390/ijms21207538 PMC7589924 33066082

[B30] Pulvirenti G. Parisi G. F. Giallongo A. Papale M. Manti S. Savasta S. (2019). Lower Airway Microbiota. Front. Pediatr. 7, 393. 10.3389/fped.2019.00393 31612122 PMC6776601

[B31] Ramos-Sevillano E. Wade W. G. Mann A. Gilbert A. Lambkin-Williams R. Killingley B. (2019). The Effect of Influenza Virus on the Human Oropharyngeal Microbiome. Clin. Infect. Dis. 68, 1993–2002. 10.1093/cid/ciy821 30445563 PMC6541733

[B32] Segal L. N. Clemente J. C. Tsay J. C. Koralov S. B. Keller B. C. Wu B. G. (2016). Enrichment of the Lung Microbiome with Oral Taxa Is Associated with Lung Inflammation of a Th17 Phenotype. Nat. Microbiol. 1, 16031. 10.1038/nmicrobiol.2016.31 27572644 PMC5010013

[B33] Sze M. A. Dimitriu P. A. Suzuki M. McDonough J. E. Campbell J. D. Brothers J. F. (2015). Host Response to the Lung Microbiome in Chronic Obstructive Pulmonary Disease. Am. J. Respir. Crit. Care Med. 192, 438–445. 10.1164/rccm.201502-0223OC 25945594 PMC4595667

[B34] Tsay J. J. Wu B. G. Badri M. H. Clemente J. C. Shen N. Meyn P. (2018). Airway Microbiota Is Associated with Upregulation of the Pi3k Pathway in Lung Cancer. Am. J. Respir. Crit. Care Med. 198, 1188–1198. 10.1164/rccm.201710-2118OC 29864375 PMC6221574

[B35] Wang J. Ding L. Zhou J. Ma H. Wu Y. Wang J. (2020). Target Lipidomics Approach to Reveal the Resolution of Inflammation Induced by Chinese Medicine Combination in Liu-Shen-Wan against Realgar Overexposure to Rats. J. Ethnopharmacol 249, 112171. 10.1016/j.jep.2019.112171 31442622

[B36] Wang Z. Locantore N. Haldar K. Ramsheh M. Y. Beech A. S. Ma W. (2021). Inflammatory Endotype-Associated Airway Microbiome in Chronic Obstructive Pulmonary Disease Clinical Stability and Exacerbations: A Multicohort Longitudinal Analysis. Am. J. Respir. Crit. Care Med. 203, 1488–1502. 10.1164/rccm.202009-3448OC 33332995 PMC8483235

[B37] Yan F. Wen Z. Wang R. Luo W. Du Y. Wang W. (2017). Identification of the Lipid Biomarkers from Plasma in Idiopathic Pulmonary Fibrosis by Lipidomics. BMC Pulm. Med. 17, 174. 10.1186/s12890-017-0513-4 29212488 PMC5719761

[B38] Yang X. Li H. Ma Q. Zhang Q. Wang C. (2018). Neutrophilic Asthma Is Associated with Increased Airway Bacterial burden and Disordered Community Composition. Biomed. Res. Int. 2018, 9230234. 10.1155/2018/9230234 30105264 PMC6076954

[B39] Yun Y. Srinivas G. Kuenzel S. Linnenbrink M. Alnahas S. Bruce K. D. (2014). Environmentally Determined Differences in the Murine Lung Microbiota and Their Relation to Alveolar Architecture. PLoS One 9, e113466. 10.1371/journal.pone.0113466 25470730 PMC4254600

[B40] Zhang C. Zhang T. Lu W. Duan X. Luo X. Liu S. (2020). Altered Airway Microbiota Composition in Patients with Pulmonary Hypertension. Hypertension 76, 1589–1599. 10.1161/HYPERTENSIONAHA.120.15025 32921193

[B41] Zhang L. Zhu B. Zeng Y. Shen H. Zhang J. Wang X. (2020). Clinical Lipidomics in Understanding of Lung Cancer: Opportunity and challenge. Cancer Lett. 470, 75–83. 10.1016/j.canlet.2019.08.014 31655086

[B42] Zhang W. Song Y. Chai T. Liao G. Zhang L. Jia Q. (2020). Lipidomics Perturbations in the Brain of Adult Zebrafish (danio Rerio) after Exposure to Chiral Ibuprofen. Sci. Total Environ. 713, 136565. 10.1016/j.scitotenv.2020.136565 31954244

[B43] Zhang F. Wan Y. Zuo T. Yeoh Y. K. Liu Q. Zhang L. (2021). Prolonged Impairment of Short-Chain Fatty Acid and L-Isoleucine Biosynthesis in Gut Microbiome in Patients with Covid-19. Gastroenterology 277, 1–10. 10.1053/j.gastro.2021.10.013 PMC8529231 34687739

[B44] Zhao J. Wang Y. Huang X. Ma Q. Song J. Wu X. (2021). Liu Shen Wan Inhibits Influenza Virus-Induced Secondary staphylococcus Aureus Infection *In Vivo* and *In Vitro* . J. Ethnopharmacol 277, 114066. 10.1016/j.jep.2021.114066 33766755

